# The effects on coagulation of the reinfusion of unprocessed residual blood from the cardiopulmonary bypass

**DOI:** 10.1186/s13104-016-1868-y

**Published:** 2016-02-03

**Authors:** Yolande-Leigh Iyer, Philip Hayward, Larry McNicol, Laurence Weinberg

**Affiliations:** Department of Cardiac Surgery, Austin Hospital, Heidelberg, VIC Australia; Department of Surgery, University of Melbourne, Parkville, VIC Australia; Department of Anaesthesia, Austin Hospital, Studley Road, Heidelberg, VIC 3084 Australia; Department of Surgery and Centre for Anaesthesia, Perioperative and Pain Medicine, University of Melbourne, Parkville, VIC Australia

**Keywords:** Blood salvage, Autologous blood transfusion, Cardiopulmonary bypass residual blood, Thromboelastography

## Abstract

**Background:**

Autologous blood transfusion is a common technique in cardiac surgery to directly re-infuse residual blood from the cardiopulmonary bypass (CPB) circuit to the patient. The objective of this study was to evaluate the effects of reinfusion of unprocessed residual pump blood on the coagulation system after separation from the CPB circuit and reversal of systemic heparin with protamine.

**Measurements and main results:**

After ethics approval, 40 participants undergoing cardiac surgery were recruited in a prospective single center cohort study. Changes in coagulation were assessed with standard plasma based laboratory assays and thromboelastography. After the reinfusion of unprocessed residual pump blood there were decreases in the mean aPTT (effect size 6 s; SD: 6.05; p < 0.0001) and thrombin time (effect size 4.08 s; SD: 9.7; p = 0.01). There were no significant changes in PT, INR and D-dimer. Post reinfusion there were increases in fibrinogen, hemoglobin and platelet counts. There were improvements in the R-time (effect size 9.1 s; SD: 16.9; p = 0.0023), K-time (effect size 1.5 s; SD: 3.6 s; p = 0.0017), alpha angle (6.9°; SD: 15.8; p = 0.012), and maximum amplitude (3.0 mm; SD: 5.6, p = 0.002) on thromboelastography.

**Conclusion:**

The reinfusion of unprocessed residual CPB blood resulted in no deleterious effects on the coagulation system measured by both the common laboratory plasma based measurements of coagulation and thromboelastography.

## Background

In cardiac surgery, the transfusion of red blood cells is considered beneficial in situations in which a critically low hematocrit may contribute to inadequate tissue oxygen delivery. Risks associated with transfusion of allogeneic blood include bacterial and viral contamination, transfusion-associated lung injury, and transfusion-associated immunological risks including incompatibility of a transfusion with donor ABO group antigens, allo-immunization; immunization with the HLA group antigens and transfusion-mediated immunomodulation [1]. In cardiac surgery, autologous transfusion (collection and reinfusion of the patient’s own blood) is therefore a common technique. This may involve reinfusion of washed red blood cells after collection using a cell salvage device. However, a more widely used technique is the direct reinfusion of unprocessed (unwashed) residual blood from the cardiopulmonary bypass (CBP) circuit after separation from CPB and reversal of systemic heparin with protamine. The advantages of this include increased hematocrit and hemoglobin values [2], greater thrombin generation, fibrinolysis activation and lower fibrinolysis inhibition [3]. However, as the unprocessed residual blood from the CPB is heparinised, it is a common assumption that a “re-heparin” effect frequently develops with adverse effects on the coagulation system. Therefore we hypothesised that the reinfusion of heparinised autologous blood administered after separation from the CPB circuit and complete reversal of systemic heparin with protamine, leads to prolongation of the common laboratory plasma based measurement of coagulation and derangements of the normal viscoelastic clotting properties of blood as measured by thromboelastography.

## Methods

### Participants

The Austin Health Research and Ethics Committee approved this study (number: LNR/13/Austin/291/2013) and all participants gave written informed consent. Between January 2013 and June 2013 participants were recruited from perioperative anesthesia and cardiac clinics at the Austin Hospital, a large University-affiliated, metropolitan hospital in Melbourne, Australia. Inclusion criteria included adult patients (>18 years) undergoing either primary coronary artery bypass graft or valve surgery requiring CPB. Exclusion criteria included a history of bleeding disorders, any preoperative abnormality in the common laboratory plasma based measurement of coagulation, thrombocytopenia, abnormal liver function tests, redo surgery, history of endocarditis, and preoperative use within a 10-day period of any of the following medications; warfarin, heparin (high or low molecular weight), adenosine diphosphate receptor inhibitors, glycoprotein IIB/IIIA inhibitors, adenosine re-uptake inhibitors or thromboxane inhibitors.

### Primary outcomes

The primary aim was to evaluate changes in coagulation after the reinfusion of the unprocessed residual heparinised blood from the CPB circuit administered after separation from the CPB circuit and complete reversal of systemic heparin with protamine. Standard plasma based laboratory assays of coagulation were used to measure aPTT, prothrombin time (PT), thrombin time (TT), D-dimer, and fibrinogen. As these standard laboratory assays are often considered to be poor predictors of bleeding and thrombosis, thromboelastography was used to measure the viscoelastic properties of whole blood clot formation. Hemoglobin (by absorption spectrophotometry) and platelet count (using impedance methodology with hydrodynamic focusing) were also measured using a Sysmex XE5000 analyser (Sysmex corporation, Japan). Laboratory coagulation tests were performed using an ACL TOP 500 analyser (Instrumentation Laboratory, USA). The PT was measured using a clot based assay using recombinant tissue factor relipidated in a synthetic phospholipid blend combined with calcium chloride (PT-Recombiplastin reagent, Instrumentation Laboratory, USA). Fibrinogen was derived from absorbance during the PT clot based assay relative to a calibrator (PT-Recombiplastin reagent, Instrumentation Laboratory, USA). The TT used a clot based assay using purified bovine thrombin to convert fibrinogen to fibrin (Thrombin Time reagent, Instrumentation Laboratory, USA). D-Dimer was measured using a latex enhanced immunoassay (D-Dimer HS reagent, Instrumentation Laboratory, USA). The aPTT was measured using clot based assay using purified phospholipid and micronized silica activator (Triniclot HS reagent, TCOAG, Ireland). The viscoelastic properties of whole blood clot formation were measured on a TEG^®^ 5000 Hemostasis Analyzer System (Hemonetics^®^, USA) as per standard hospital protocols.

### Standardization of setting

General anesthesia was managed by a group of cardiac anesthesiologists using a standardised care protocol. The study was conducted in a dedicated cardiac theatre with standardization of ambient air temperature set to 21 °C (69.8 F). All patients were fasted 6-h for solids and 2-h for clear fluids. Prior to induction of anesthesia there was no additional intravenous fluid administered. Induction of anesthesia consisted of a standard technique using propofol (0.5–1 mg/kg), fentanyl (5–10 μg/kg) and a non-depolarizing neuromuscular blocker. Maintenance of anesthesia was achieved using sevoflurane or isoflurane in 50 % oxygen: 50 % air ratio titrated to a bispectral index (BIS) of 40–60. No patient received any antifibrinolytic therapy (e.g. tranexamic acid). Intra-operative autologous hemodilution was not utilised as part of the anesthesia technique for any patient. Prior to initiation of CPB fluid intervention was restricted but if clinically indicated, a balanced crystalloid solution was used. No colloid solutions were used in the pre CPB period. Prior to commencement of CPB heparin (300 U/kg) was administered to achieve a therapeutic activated clotting time (ACT) of greater than 480 s. If ACT was sub-therapeutic repeated boluses of heparin (5000 U) were administered. The CPB circuit was primed with 10,000 U heparin. For the pump prime crystalloid solution, all patients received 2000 ml of Plasma-Lyte 148^®^ (Baxter, Sydney, NSW). Cardiopulmonary bypass was performed using a membrane oxygenator (Quadrox-i, Maquet Cardiopulmonary, Hirrlingen, Germany). The pump rate was set at 2.4 l m^−2^ min and body temperature was maintained between 32 and 34 °C (89.6–93.2 F). A standard induction cardioplegia solution (Baxter Viaflex^®^, AHK 5560, Toongabie, NSW) followed by a maintenance cardioplegia solution (Baxter Viaflex^®^ AHK6029, Toongabie, NSW) was administered to all patients.

During CPB the ACT was maintained above 480 s with additional boluses of heparin if required. If volume supplementation was required during CPB, fluid boluses of Plasma-Lyte 148 were used. The use of additional blood products was at the discretion of the attending clinical perfusionist and anesthesiologist. Normothermia was maintained in all patients after separation from CPB by using forced air warming devices and a fluid warmer ensuring delivery of fluid at 42 °C (107.6 F). At the termination of CPB, 750 ml of unprocessed residual blood remaining within the reservoir and tubing of the CPB circuit was collected and transferred to a standardised non-citrated, 1000 ml sterile clear bag (Baxter Healthcare, Toongabbie, NSW) over a 5-min period. Systemic heparin was reversed with a standardised dose of protamine (1.5 mg/kg for very 100 U of heparin) to achieve an ACT of less than 150 s. If ACT was above 150 s, additional protamine (50 mg) was titrated in small aliquots. When the ACT was less than 150 s, the unprocessed residual blood was then reinfused into the patient over a 15-min period.

Blood was sampled from the patient at two key time points to evaluate changes in coagulation before and after the re-infusion of the unprocessed residual blood.

1. 5 min prior to the reinfusion of the unprocessed residual blood.
2. 15 min after the reinfusion of the unprocessed residual blood.

Sampling was performed by aspirating blood from the indwelling arterial catheter using a standard 10 ml syringe (Terumo^®^ Corporation, Tokyo, Japan) syringe over a 10 s period. In order to assess for contact activation of the autologous blood from the plastic re-infusion bag, additional blood was sampled from the 750 ml unprocessed residual blood in the collection bag, and from the CPB circuit itself. All samples underwent standardised laboratory and TEG coagulation studies, which included plasma based laboratory assays of coagulation i.e. aPTT, PT, thrombin time (TT), D-dimer, and fibrinogen and platelet count, and measurements of the viscoelastic properties of whole blood clot formation using TEG. This included measurement of R-time, K-time, alpha angle, and maximum amplitude.

### Statistical analysis

The study was powered to determine whether the re-administration of unprocessed residual blood from the CPB circuit would have a residual heparin effect on the patient. An increase in aPTT by 15 % from baseline was considered clinically significant. In the context of cardiac surgery, pilot data from 63 patients undergoing cardiac surgery at our institution showed that after separation from CPB and reversal of systemic heparin with protamine (1.5 mg/kg for very 100 U of heparin) the mean aPTT was 41 s (SD: 5.0 s). We hypothesized that the administration of unprocessed residual heparinised blood would increase the aPTT by 15 % (i.e. an increase in the aPPT from 41 to 47 s). With an alpha value of 0.05 and a desired power of 0.90, 8 participants were required. In order to observe important changes in other standard plasma based laboratory assays and TEG variables, we received ethics approval to recruit 40 participants.

Statistical analysis was performed using PRISM 6 (GraphPad Software Inc., USA). All values analyzed are expressed as mean with standard deviation (ST) and median with interquartile range (IQR) depending on whether the data was normally distributed. Normality was tested using the D’Agostino & Pearson omnibus normality test and the Shapiro–Wilk normality test. Statistical comparisons between data points were done by the parametric two tailed paired t tests and non-parametric Wilcoxon Signed Rank test. All p values less than 0.05 were considered statistically significant.

## Results

Forty participants consented for the study and received the intervention i.e. reinfusion of 750 ml of unprocessed residual heparinised blood from the CPB circuit. There were no breaches in study protocol. The participant demographic data is summarized in Table 1.

**Table 1:** Patient demographic data

Demographic variable	Value
Age (years)	64 (12)
Gender (males/females)	29/11
Type of surgery (CABG/valvular)	32/8
Euroscore II	7 (3)
Height (cm)	166 (15)
Weight (kg)	81 (24)
BMI (kg/m^2^)	28 (6)
Dose of heparin (units)	25,000 (7000)
Dose of protamine (mg)	360 (110)
Total time on CPB circuit (mins)	104 (37)
Aortic clamp time (mins)	83 (32)

Data is presented as mean (standard deviation)

### Coagulation results

Results of preoperative blood samples are shown in Table 2. Results of blood samples from both the 750 ml bag unprocessed residual blood and from the CPB circuit showed a prolonged aPTT and TT in keeping with a full systemic effect of heparin on the intrinsic coagulation pathway. As heparin also affects the common coagulation pathway, the INR and PT also were also prolonged (Table 3). The key primary outcome measures are summarized in Table 4, which shows the standard laboratory coagulation, fibrinolytic and TEG variables. Post reinfusion of the unprocessed residual heparinised blood, the mean aPTT decreased from 42 to 35 s (effect size 6 s; SD: 6.05; p < 0.0001). Similarly, there were improvements of the following coagulation parameters: (1) the mean thrombin time decreased from 25 s to 21 s (effect size 4.08 s; SD: 9.7; p = 0.01), (2) mean fibrinogen increased from 1.9 to 2.0 g/l (effect size 0.1 g/l, SD: 0.2; p = 0.05). Post re-infusion there were no significant changes in PT or INR compared to pre reinfusion values (p = 0.15 and p = 0.24 respectively). There was no significant change in D-dimer post reinfusion compared to pre reinfusion within patients (p = 0.52). There was no correlation between pre-infusion R times and dose of protamine administered (Spearman correlation r = −0.09, 95 % CI −0.41 to 0.23, p = 0.55).

**Table 2:** Preoperative hematological, coagulation and thromboelastography parameters

	Value
*Laboratory coagulation parameters*	
Hemoglobin (g/l) (NR: 115–165)	126 (15)
Platelets (×10^9^/l) (NR: 150–400 × 10^9^)	190 (52)
Prothrombin time (s) (NR: 11–15)	12 (1.1)
INR (NR: <1.4)	1.1 (0.10)
aPTT (s) (NR: 22–38)	29 (5.50)
Thrombin time (s) (NR: 13–21)	18 (3.60)
Fibrinogen (g/l) (NR: 2–4)	3.1 (0.88)
D-Dimer (mg/l) (NR: <0.23)	0.24 (0.14)
*TEG parameters (heparinase)*	
R-time (min) (NR: 4–8)	6.3 (1.70)
K-time (min) (NR: 0–4)	1.6 (0.36)
Alpha angle (degrees) (NR: 47–74)	72 (3.70)
Maximum amplitude (mm) (NR: 54–72)	70 (5)
*TEG parameters (native)*	
R-time (mins) (NR: 4–8)	7.5 (5)
K-time (mins) (NR: 0–4)	2.2 (2.50)
Alpha angle (degrees) (NR: 47–74)	70 (8.60)
Maximum amplitude (mm) (NR: 54–72)	71 (5.30)

Values are mean with standard deviation unless otherwise stated

*NR* normal range

**Tables 3:** Hematological, coagulation and thromboelastography parameters for the cardiopulmonary bypass pump residual blood and transfusion bag blood

	CBP pump	Transfusion bag
*Laboratory coagulation parameters*		
Hemoglobin (g/L) (NR: 115–165)	85 (17)	80 (14)
Platelets (×10^9^/L) (NR: 150–400)	126 (45)	126 (43)
Prothrombin time (s) (NR: 11–15)	91 (44)	85 (46)
INR (NR: <1.4)	7.9 (3.40)	7.2 (3.80)
aPTT (s) (NR: 22–38)	All values >200	All values >200
Thrombin time (s) (NR: 13–21)	All values >60	All values >60
Fibrinogen (g/L) (NR: 2–4)	1.1 (0.27)	1.2 (0.29)
D-Dimer (mg/L) (NR: <0.23)	1.8 (1.90)	2 (2.70)
*TEG parameters (heparinase)*		
R-time (mins) (NR: 4–8)	7.1 (2.40)	6.8 (4.60)
K-time (mins) (NR: 0–4)	2.4 (3)	0.75 (1.90)
Alpha angle (degrees) (NR: 47–74)	70 (7.7)	70 (6.90)
Maximum amplitude (mm) (NR: 54–72)	60 (9.10)	59 (7)

Values are mean with standard deviation unless otherwise stated

*NR* normal range

**Table 4:** Hematological, coagulation and thromboelastography parameters pre and post infusion of autologous residual blood

	Pre infusion	Post infusion	p value
*Laboratory coagulation parameters*			
Hemoglobin (g/l) (NR: 115–165)	89 (14)	93 (11)	0.003*
Platelets (×10^9^/l) (NR: 150–400)	96 (34)	118 (39)	0.0007*
*Laboratory coagulation parameters*			
Prothrombin time (s) (NR: 11–15 s)	18	17 (3.30)	0.15
INR (NR: <1.4)	1.6	1.5 (0.29)	0.24
aPTT (s) (NR: 22–38)	42 (20)	35 (6.4)	<0.0001*
Thrombin time (s) (NR: 13–21)	25 (10)	21 (5.5)	0.01*
Fibrinogen (g/l) (NR: 2–4)	1.9 (0.51)	2 (0.56)	0.05*
D-Dimer (mg/l) (NR: <0.23)	1.5 (1.60)	1.6 (1.60)	0.52
*TEG parameters (heparinase)*			
R-time (min) (NR: 4–8)	21 (6)	12 (9)	0.0023*
K-time (min) (NR: 0–4)	3.9 (3.30)	1.5 (2.50)	0.0017*
Alpha angle (degrees) (NR: 47–74)	59 (14)	65 (10)	0.012*
Maximum amplitude (mm) (NR: 54–72)	60 (8.30)	62 (7)	0.002*
*TEG parameters (native)*			
R-time (mins) (NR: 4–8)	26 (24)	13 (10)	0.0041*
K-time (mins) (NR: 0–4)	5 (5.50)	2.5 (2)	0.012*
Alpha Angle (degrees) (NR: 47–74)	58 (15)	67 (9.40)	0.0051*
Maximum amplitude (mm) (NR: 54–72)	61 (11)	65 (7)	0.0078*

Values are mean and standard deviation

*NR* normal range

*p < 0.05 are statistically significant

### Hematological parameters

Post reinfusion of the unprocessed residual heparinised blood there were increases in mean hemoglobin from 89 to 93 g/l (effect size 4.85 g/l; SD: 0.69; p = 0.003) and platelet count increased from 96 × 10^9^/l to 118 × 10^9^/l (effect size 18 × 10^9^/l; SD: 31.2; p = 0.0007).

### Thromboelastography parameters

Native and heparinase TEG parameters are summarized in Table 4. For the heparinase samples, after the reinfusion of the ununprocessed residual blood, the R-time (time taken for initial fibrin formation) decreased from 21 to 12 min (effect size 9.1 min; SD: 16.9; p = 0.0023), K-time (speed of clot formation) decreased from 3.9 to 1.5 min (effect size 1.5 min. SD: 3.6 min; p = 0.0017), alpha angle (rate of clot formation) increased from 59° to 65° (effect size: 6.9°; SD: 15.8; p = 0.012) and MA increased from 60 mm to 62 mm (effect size 3.0 mm; SD: 5.6, p = 0.002). There were similar improvements in all the native TEG samples, which are summarized in Table 4.

### Outcomes

The mean blood loss from the mediastinal chest drains at 24 h post-operatively was 1024 ml (SD 918). Fourteen patients (35 %) received a packed red blood cell transfusion. The median number of units transfused was 2 (IQR 1:1.25). The mean volume of blood transfused was 585 ml (SD 447 ml). Five participants (13 %) received post-operative platelet transfusions. The median number of pooled platelets transfused was 1 unit (IQR 1:3) Six patients (15 %) received post-operative Fresh Frozen Plasma transfusions. The median number of bags of Fresh Frozen Plasma transfused was 2 (IQR 1.75:5.75). Four patients (10 %) received post-operative cryoprecipitate transfusions. The median number of units of cryoprecipitate transfused was 10 (IQR 7:10). There was no correlation between postoperative 24-h mediastinal drainage and post-infusion R times (Spearman correlation r = −0.004, 95 % CI −0.33 to 0.32, p = 0.98). The median duration of ICU stay was 22 h (IQR 20:42.5). The median length of hospital stay was 8.6 days (IQR 8:10).

## Discussion

### Key study findings

We performed a prospective observational study on forty patients undergoing cardiac surgery requiring CPB, evaluating the effect of reinfusion of 750 ml of residual unprocessed heparinised blood from the CPB circuit on coagulation. The reinfusion of unprocessed blood increased hemoglobin concentration and platelet count, but contrary to the hypothesis resulted in improvements of the prolonged common laboratory plasma based measurements of coagulation (aPTT, Thrombin Time, fibrinogen concentration). Furthermore, post reinfusion there were improvements in the viscoelastic clotting properties of blood measured by TEG (R-time, K-time, alpha angle, MA).

After the administration of protamine and before the reinfusion of the unprocessed residual heparinised blood, a post CPB coagulopathy was present as evident by prolongation of common laboratory based measures of coagulation and prolonged derangements of both the native and heparinase TEG values. Post CPB coagulopathy can be caused by a combination of haemodilution, coagulation factor depletion and platelet dysfunction, fibrinolysis and residual heparin [4]. We consider a residual heparin effect to be very unlikely as the TEG R-time did not correct with heparinase. Furthermore, there was also no TEG evidence of fibrinolysis or platelet dysfunction. As the unprocessed residual blood from the CPB circuit is heparinized, we expected the coagulation parameters to worsen. Contrary to our hypothesis, this effect was not observed. Rather, after administration of the residual blood there were improvements in the patient’s laboratory and TEG parameters of coagulation. The hypothesis of excess protamine effect post CPB is also supported by the observation that the mean R time (heparinase) in the pre infusion sample was greater than the R time in the CPB circuit blood. This is unlikely to be due to haemodilution, as there was not a substantial amount of fluid given in the immediate post CPB period. Likely, the only significant difference between the two time points was the administration of protamine. Further evidence to support a protamine effect could have been obtained by performing anti-Xa assays. Unfortunately, frozen plasma samples were not retained to carry out further testing.

We attribute the post CPB coagulopathy observed in our study to be a protamine effect. Adverse affects of clot initiation time, kinetics and platelet function from protamine have been previously described [5]. Generally it is assumed that 1 mg of protamine neutralizes 100 U of heparin. In our study, a higher dose of protamine was administered (1.5 mg/kg for very 100 U of heparin). Therefore, the cause of the post bypass coagulopathy may be readily attributed to an excess protamine effect. Interestingly, the improvements in the aPTT observed after the administration of the unprocessed residual heparinised CPB blood may be explained by the heparin from the unprocessed residual blood neutralizing or “mopping up” excess or residual protamine, hence negating the adverse effects of protamine of the coagulation system. Protamine administration in excess of heparin has also been shown to have adverse effects on collagen-induced platelet thrombus formation in addition to impairing the platelet GPIb-vWF interaction necessary for normal hemostasis [6]. Platelet adhesion to the injured vessel wall is essential in hemostasis and thrombosis, a process that involves the interaction of the platelet glycoprotein Ib with surface bound von Willebrand factor (vWF). Since synthetic polycationic peptides of the general formula (Arg)n, (Lys)n or (Arg-Lys)n inhibit GPIb-vWF interaction, they have been suggested as potential antithrombotics. Protamine is a highly cationic polypeptide, with arginine accounting for approximately 60 % of the primary sequence, utilised to neutralise the anticoagulant effect of heparin after cardiac surgery [7].

The improvement in hemoglobin found post intervention was expected as the pump blood contains a large volume of red blood cells. The improvement in platelet count was however unexpected and whilst statistically significant, is considered of little clinical significance as the mean difference between pre and post intervention platelet count was only 18 × 10^9^/l. Since the CPB residual blood contains a small number of platelets, the overall addition of the residual blood to the patients total blood volume should lead to a mild decrease in platelet count due to a haemodilution effect.

### Relationship to previous studies

Only a limited number of studies have compared the effect of reinfusion of the unprocessed residual pump blood on coagulation and none have used both plasma based coagulation and fibrinolysis assays and thromboelastography whole blood analysis. Daane et al. [8] found impaired hemostasis and hyperfibrinolysis after the transfusion of both unprocessed and processed CPB residual, compared to preoperative levels. The impaired coagulation and hyperfibrinolysis found post CPB compared to pre CPB baseline correlated with our study. Daane et al. also found an improvement in the coagulation values as time progressed following CPB. However, limitations of their study were that the volume of residual blood transfused, and the period over which it was reinfused, was not standardized. Therefore it would be difficult to distinguish between the effects of time alone and those due to reinfusion of the residual blood from the CPB circuit. In contrast, in our study, both the volume of the residual blood transfused and the transfusion times were standardized in all patients, increasing the validity our findings.

The effects of CPB residual blood content was further studied by Flom-Halvorsen et al. [9] who evaluated the hemostatic and inflammatory markers in 40 patients undergoing CPB. The hemostatic and immunologic systems were moderately activated in the autologous blood remaining in the extracorporeal circuit, whereas the mediastinal shed blood was highly activated in all aspects. Autotransfusion had no correlating clinical side-effects and the subsequent exposure to allogeneic blood products was minimal. Similar to the findings of our study, Flom-Halvorsen et al. observed that blood from both the unprocessed residual pump blood bag and bypass circuit were analyzed and found to have globally impaired coagulation. As the unprocessed residual CPB blood is heparinized, it may have adverse effects on the intrinsic and common coagulation pathways. Thrombin time is also sensitive to heparin and was also prolonged in our study, again suggestive of a heparin effect. Finally, Campbell et al. [10] evaluated the impact of cell salvage during cardiac surgery on the thromboelastomeric coagulation profiles. Residual CPB volume was processed and transfused after surgery in the cell salvage group and the residual volume was transfused unprocessed in the control group. Similar to our findings, there was an increase in the clotting time (K-time) and clot formation time (R-time) in patients post separation from bypass compared to baseline values. Cell salvage of the residual CPB volume reduced platelet numbers and prolonged clot formation time and maximum clot firmness. However, unlike our study comparisons were made with pre surgical values and not immediate pre transfusion values.

### Strengths and limitations

Our study also has several methodological strengths. Information on detailed changes in both the standard plasma based assays for coagulation and TEG were performed supporting the validity of our findings. Analysis of the blood from the CPB circuit in addition to the unprocessed heparinised blood from the transfusion bag has further strengthened the internal validity of the study. This ensured there were no additional study confounders and all patients were infused with unprocessed heparinised residual blood with similar laboratory coagulation parameters. Standardization of the doses of heparin and protamine ensured consistency between patients, and a more accurate interpretation of our findings. Although our institution is a major tertiary teaching hospital with a high caseload of complex cardiac procedures, it is not possible to generalize the results of this study to other centers, different types of cardiac surgery, or centres that use different heparin and protamine protocols. This is a single centre study, which may limit the external validity of our findings. However our hospital has all the typical characteristics of a tertiary institution in a developed country and a recent comparative study confirmed that its patients and their outcomes were equivalent to those of other tertiary hospitals in Australia [11].

There are also several limitations to our study. First, the trial was small and only powered to show changes in the aPTT. However changes in the other secondary outcomes, particularly the TEG variables are important and allow a greater understanding of the changes observed. There is potential for other confounders to have influenced the outcomes measured. In particular, cardiac surgery involves several heterogeneous surgical techniques, pathologies and patients, which may not have been exactly similar in our small sample size. Participants were used as their own controls, and the study design could have been strengthened by randomizing patients to compare those that received unprocessed residual CPB blood and those that did not. Finally, further tests for special markers of coagulation activation (e.g. prothrombin fragments, plasmin-anti-plasmin, plasminogen activator inhibitor-1, and interleukin-6) were not be carried out, which may have provided further information about the effects of pump blood on coagulation activation.

## Conclusion

This study demonstrates that after separation from CPB and systemic heparin reversal with protamine, the reinfusion of 750 ml of unprocessed heparinised blood from the CPB circuit improved hemoglobin concentration and platelet counts. Contrary to the findings of other studies [8–10], reinfusion of the unprocessed heparinised blood resulted in no adverse effects on the coagulation system measured by both the common laboratory plasma based measurements of coagulation and thromboelastrography.
